# Impact of *Campylobacter jejuni cj0268c* Knockout Mutation on Intestinal Colonization, Translocation, and Induction of Immunopathology in Gnotobiotic IL-10 Deficient Mice

**DOI:** 10.1371/journal.pone.0090148

**Published:** 2014-02-25

**Authors:** Markus M. Heimesaat, Raimond Lugert, André Fischer, Marie Alutis, Anja A. Kühl, Andreas E. Zautner, A. Malik Tareen, Ulf B. Göbel, Stefan Bereswill

**Affiliations:** 1 Department of Microbiology and Hygiene, Charité - University Medicine Berlin, Berlin, Germany; 2 Department of Medical Microbiology, University Medical Center Göttingen, Göttingen, Germany; 3 Department of Pathology/Research Center ImmunoSciences (RCIS), Charité - University Medicine Berlin, Berlin, Germany; 4 Department of Clinical Chemistry/UMG-Laboratory, University Medical Center Göttingen, Göttingen, Germany; Indian Institute of Science, India

## Abstract

**Background:**

Although *Campylobacter jejuni* infections have a high prevalence worldwide and represent a significant socioeconomic burden, the underlying molecular mechanisms of induced intestinal immunopathology are still not well understood. We have recently generated a *C. jejuni* mutant strain NCTC11168::*cj0268c*, which has been shown to be involved in cellular adhesion and invasion. The immunopathological impact of this gene, however, has not been investigated *in vivo* so far.

**Methodology/Principal Findings:**

Gnotobiotic IL-10 deficient mice were generated by quintuple antibiotic treatment and perorally infected with *C. jejuni* mutant strain NCTC11168::*cj0268c*, its complemented version (NCTC11168::*cj0268c*-comp-*cj0268c*), or the parental strain NCTC11168. Kinetic analyses of fecal pathogen loads until day 6 post infection (p.i.) revealed that knockout of *cj0268c* did not compromise intestinal *C. jejuni* colonization capacities. Whereas animals irrespective of the analysed *C. jejuni* strain developed similar clinical symptoms of campylobacteriosis (i.e. enteritis), mice infected with the NCTC11168::*cj0268c* mutant strain displayed significant longer small as well as large intestinal lengths indicative for less distinct *C. jejuni* induced pathology when compared to infected control groups at day 6 p.i. This was further supported by significantly lower apoptotic and T cell numbers in the colonic mucosa and lamina propria, which were paralleled by lower intestinal IFN-γ and IL-6 concentrations at day 6 following knockout mutant NCTC11168::*cj0268c* as compared to parental strain infection. Remarkably, less intestinal immunopathology was accompanied by lower IFN-γ secretion in *ex vivo* biopsies taken from mesenteric lymphnodes of NCTC11168::*cj0268c* infected mice versus controls.

**Conclusion/Significance:**

We here for the first time show that the *cj0268c* gene is involved in mediating *C. jejuni* induced immunopathogenesis *in vivo*. Future studies will provide further deep insights into the immunological and molecular interplays between *C. jejuni* and innate immunity in human campylobacteriosis.

## Introduction

*Campylobacter jejuni* is the most important cause of bacterial diarrhea in developing as well as in industrialized countries. The characteristic features of the disease vary from watery to bloody diarrhea accompanied by abdominal cramps and fever. In rare cases complications such as the Guillain-Barré syndrome might arise post infection (p.i.) [1], [2]. Although many virulence factors of *C. jejuni* have been described yet, the overall image of this bacterial infection is still incomplete [3], [4].

A successful infection with *C. jejuni* requires adherence of the pathogen to host cells and several proteins of *C. jejuni* that contribute to this initial interaction have been characterized in the past. MOMP, CadF and FlpA, for instance, were shown to possess fibronectin-binding properties whereby specifically CadF and FlpA initiate the remodelling of the actin cytoskeleton *via* the activation of integrin receptors to allow internalization of *C. jejuni* into the host cell [5], [6], [7]. Furthermore, PEB1 as an element of an ABC transporter and CapA, representing an autotransporter protein, mediate adherence and are important for *C. jejuni* colonization of mice and chicken, respectively [8], [9]. Cj0091 and JlpA are additionally necessary for the adherence of *C. jejuni* to host cells whereby a JlpA-HSP 90alpha interaction is going along with the activation of NF-κB and the p38 MAP kinase [10], [11], [12]. Complementary to the proteins described above, lipooligosaccharides (LOS) contribute to the adherence properties since a *C. jejuni* strain deficient in LOS metabolism possesses a significantly reduced interaction with chicken embryo fibroblasts [13]. Furthermore, we characterized a *C. jejuni* mutant, which lacks a functional sulphite:cytochrome c oxidoreductase (SOR) leading to a diminished transcription of genes involved in legionaminic acid synthesis and a reduced adherence to Caco2 cell [14], [15].

Recently, we investigated the *in vitro* properties of *C. jejuni* protein Cj0268c, which has been shown by our and other groups to be important for the invasion of Caco2 cells by the pathogen [15], [16], [17]. Thereby, we could show that the invasion-relevant phenotype of Cj0268c is due to its adherence mediating function, not only in *C. jejuni* but also when this protein is expressed heterologously in *E. coli* [17]. However, the functional relevance of Cj0268c for the interaction of *C. jejuni* with the host immune system has not been demonstrated so far.

To address this we here applied the gnotobiotic murine IL-10^−/−^ infection model. In order to eradicate the colitogenic stimuli derived from the conventional intestinal microbiota, IL-10^−/−^ mice were subjected to a broad-spectrum antibiotic treatment for at least 3 months starting immediately after weaning [18]. Upon *C. jejuni* infection gnotobiotic IL-10^−/−^ mice get readily colonized by the pathogen and display acute enterocolitis within one week p.i.mimicking severe campylobacteriosis in humans, whereas gnotobiotic or with human microbiota reassociated wildtype mice display intestinal pro-inflammatory immune responses but no overt clinical symptoms such as bloody diarrhea upon *C. jejuni* infection [18]. We here for the first time investigated i) the colonization capacities and ii) clinical as well as iii) intestinal pro-inflammatory immune cell and cytokine responses upon infection of gnotobiotic IL-10^−/−^ mice with the *C. jejuni* mutant strain NCTC11168::*cj0268c*, its complemented version NCTC11168::*cj0268c*-comp-*cj0268c* and the parental strain NCTC11168.

## Results

### Impact of Cj0268c on *C. jejuni* Colonization Capacity in Infected Gnotobiotic IL-10^−/−^ Mice

**Figure 1:**
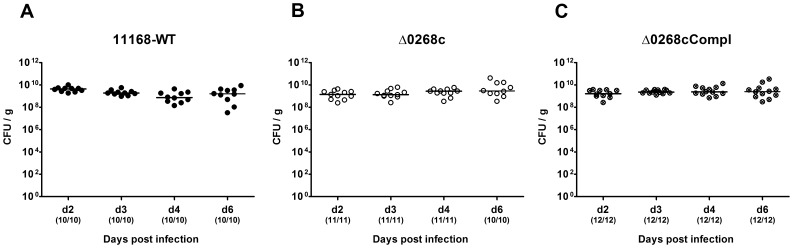
Kinetic survey of *C. jejuni* knockout mutant NCTC11168::*cj0268c* colonization in gnotobiotic IL-10^−/−^ mice. Gnotobiotic IL-10^−/−^ mice were generated by antibiotic gut decontamination and perorally infected with *C. jejuni* NCTC11168 (11168-WT, closed circles; **A**), mutant strain NCTC11168::*cj0268c* (Δ0268c, open circles; **B**), or the complemented strain NCTC11168::*cj0268c*-comp-*cj0268c* (Δ0268cCompl, crossed circles; **C**) as described (see methods). The intestinal colonization capacities over time were determined by quantification of live *C. jejuni* in fecal samples applying cultural analysis (CFU, colony forming units) starting two days until six days post infection as indicated on the x-axis. Medians (black bars) are indicated and numbers of animals harbouring the respective *C. jejuni* strain out of the total number of analyzed animals given in parentheses. Data shown were pooled from three independent experiments.

**Figure 2:**
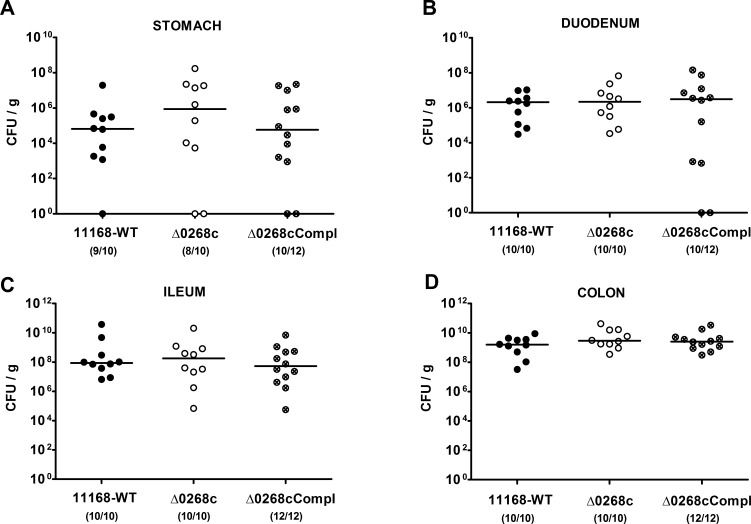
*C. jejuni* knockout mutant NCTC11168::*cj0268c* colonization along the gastrointestinal tract of gnotobiotic IL-10^−/−^ mice. Gnotobiotic IL-10^−/−^ mice were generated by antibiotic gut decontamination and perorally infected with *C. jejuni* NCTC11168 (11168-WT, closed circles), mutant strain NCTC11168::*cj0268c* (Δ0268c, open circles), or the complemented strain NCTC11168::*cj0268c*-comp-*cj0268c* (Δ0268cCompl, crossed circles) as described (see methods). The pathogen densities in distinct compartments of the gastrointestinal tract were determined by quantification of live *C. jejuni* in luminal samples taken from stomach, duodenum, ileum, and colon at day 6 p.i. by cultural analysis (CFU, colony forming units). Medians (black bars) are indicated and numbers of animals harbouring the respective *C. jejuni* strain out of the total number of analyzed animals given in parentheses. Data shown were pooled from three independent experiments.

Given that the murine commensal gut microbiota is essential for the physiological host resistance against *C. jejuni* infection [19], we generated gnotobiotic IL-10^−/−^ mice by quintuple antibiotic treatment for at least 3 months (refer to [20], [21]) to investigate the colonization capacity of *C. jejuni* mutant strain NCTC11168::*cj0268c*. Following peroral infection on two consecutive days with a comparable challenge of 10^9^ viable mutant *C. jejuni* NCTC11168::*cj0268c*, its complemented version NCTC11168::*cj0268c*-comp-*cj0268c*, or the parental strain NCTC11168, each in the stationary phase (not shown), gnotobiotic mice were readily colonized with comparably high loads of 10^9^ to 10^10^ colony forming units (CFU) of either strain per g feces over time until day 6 p.i. (**Fig. 1**). In addition, when luminal samples were taken from the entire gastrointestinal (GI) tract on the day of necropsy (day 6 p.i.), either *C. jejuni* strain could be cultured from the stomach, duodenum, ileum and colon, with the highest loads in the large intestine of approximately 10^9^ to 10^10^ CFU per g luminal content (**Fig. 2**). Thus, deficiency of the *cj0268c* gene did not impact gastrointestinal colonization capacities of *C. jejuni* in gnotobiotic IL-10^−/−^ mice upon peroral infection.

### Impact of Cj0268c on Clinical Symptoms in *C. jejuni* Infected Gnotobiotic IL-10^−/−^ Mice

**Figure 3:**
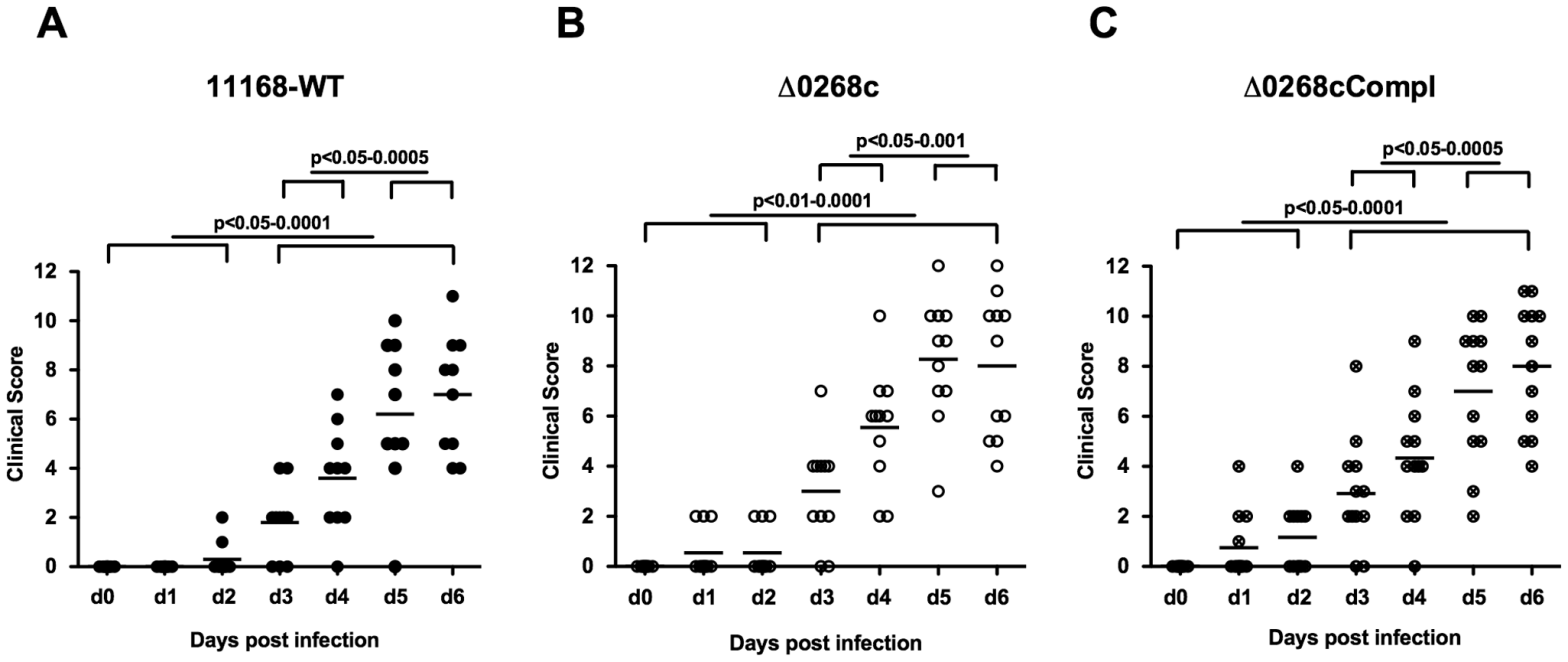
Kinetic survey of clinical symptoms following *C. jejuni* knockout mutant NCTC11168::*cj0268c* infection of gnotobiotic IL-10^−/−^ mice. Gnotobiotic IL-10^−/−^ mice were generated by antibiotic gut decontamination and perorally infected with *C. jejuni* NCTC11168 (11168-WT, closed circles, n = 10; A), mutant strain NCTC11168::*cj0268c* (Δ0268c, open circles, n = 11; B), or the complemented strain NCTC11168::*cj0268c*-comp-*cj0268c* (Δ0268cCompl, crossed circles; n = 12; C) as described (see methods). Disease activity before and following *C. jejuni* infection was assessed daily by applying a standardized clinical scoring system. Means (black bars) and levels of significance (*P*-values) determined by the Mann-Whitney-U test are indicated. Data shown were pooled from three independent experiments.

We were next interested whether a knockout of the *cj0268c* gene impacts induction of immunopathology in gnotobiotic IL-10^−/−^ mice. Daily survey of clinical conditions revealed that irrespectively whether mice had been infected with the *C. jejuni* parental strain NCTC11168, the mutant strain NCTC11168::*cj0268c* or its complemented version NCTC11168::*cj0268c*-comp-*cj0268c*, similar intestinal colonization densities were accompanied by comparable disease symptoms of enterocolitis as indicated by similar clinical scores over time (**Fig. 3**). Overt clinical symptoms started to occur around day 3 p.i. with either strain and progressed further over time reaching maximum scores at day 5 and 6 p.i. (**Fig. 3**). Notably, clinical scores of mice infected with respective *C. jejuni* strains at defined time points did not differ.

**Figure 4:**
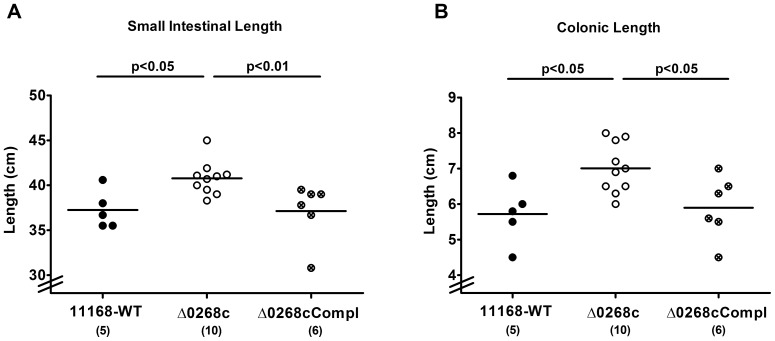
Intestinal lengths following *C. jejuni* knockout mutant NCTC11168::*cj0268c* infection of gnotobiotic IL-10^−/−^ mice. Gnotobiotic IL-10^−/−^ mice were generated by antibiotic gut decontamination and perorally infected with *C. jejuni* NCTC11168 (11168-WT, closed circles), mutant strain NCTC11168::*cj0268c* (Δ0268c, open circles), or the complemented strain NCTC11168::*cj0268c*-comp-*cj0268c* (Δ0268cCompl, crossed circles) as described (see methods). Six days following *C. jejuni* strain infections, (C) small as well as (D) large intestinal lengths (in cm) were measured at necropsy. Means (black bars), levels of significance (*P*-values) as compared to the respective control group (determined by the Mann-Whitney-U test), and numbers of analyzed animals (in parentheses) are indicated. Data shown were pooled from three independent experiments.

Given that acute intestinal inflammation is accompanied by a significant shortening of the intestinal tract [18], [20], [22], we determined the absolute lengths of the small as well as large intestines at day 6 p.i. Interestingly, gnotobiotic IL-10^−/−^ mice infected with the *C. jejuni* mutant strain NCTC11168::*cj0268c* displayed longer small intestines (approximately 10% mean difference; **Fig. 4A**) and colons (approximately 20% mean difference; **Fig. 4B**) as compared to mice infected with the parental strain NCTC11168 (p<0.05) or complemented strain NCTC11168::*cj0268c*-comp-*cj0268c* (p<0.05 and p<0.01, respectively; **Fig. 4AB**) indicative for significantly less distinct intestinal pathology. Furthermore, viable bacteria of the *C. jejuni* parental strain NCTC11168 and the complemented strain NCTC11168::*cj0268c*-comp-*cj0268c* could be cultured from mesenteric lymphnodes (MLNs) in 20.00% (2 out of 10) and 8.33% (1 out of 12) of infected animals at day 6 p.i., respectively, whereas the mutant strain NCTC11168::*cj0268c* did not translocate into MLNs at all (not shown). Furthermore, virtually no pathogenic translocation to extra-intestinal compartments could be detected given that spleen, liver, kidney and cardiac blood were exclusively *C. jejuni* culture-negative (not shown). Taken together, uncompromised colonization capacities of *C. jejuni* lacking *cj0268c* were accompanied by comparable induction of gross disease (clinical symptoms of ulcerative enterocolitis). Longer small and large intestines as well as lower translocation frequencies in *C. jejuni* mutant strain NCTC11168::*cj0268c* infected gnotobiotic IL-10^−/−^ mice, however, hint towards less pronounced intestinal immunopathology caused by absence of the *cj0268c* gene.

### Impact of Cj0268c on Induction of Intestinal Pro-inflammatory Immune Responses in *C. jejuni* Infected Gnotobiotic IL-10^−/−^ Mice

**Figure 5:**
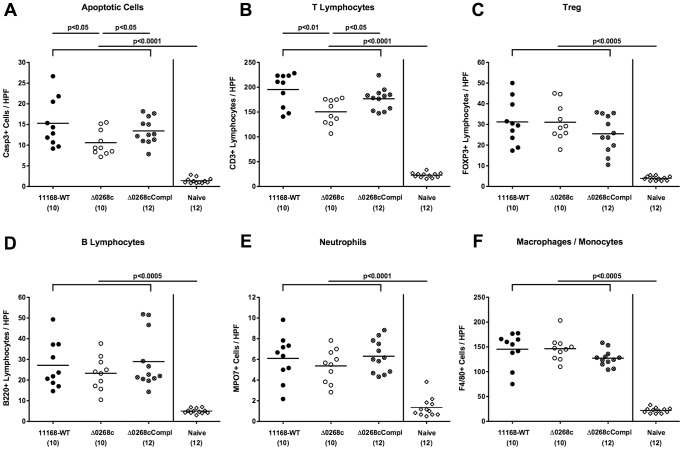
Impact of the *cj0268c* gene on colonic inflammatory and immune cell responses following infection of gnotobiotic IL-10^−/−^ mice. Gnotobiotic IL-10^−/−^ mice were generated by antibiotic gut decontamination and perorally infected with *C. jejuni* NCTC11168 (11168-WT, closed circles), mutant strain NCTC11168::*cj0268c* (Δ0268c, open circles), or the complemented strain NCTC11168::*cj0268c*-comp-*cj0268c* (Δ0268cCompl, crossed circles) as described (see methods). The average numbers of apoptotic cells (positive for caspase-3, panel A), T lymphocytes (positive for CD3, panel B), regulatory T cells (Treg, positive for Foxp3, panel C), B lymphocytes (positive for B220, panel D), neutrophils (positive for MPO7, panel E), and macrophages/monocytes (positive for F4/80, panel F) from at least six high power fields (HPF, 400x magnification) per animal were determined microscopically in immunohistochemically stained colon sections at day 6 p.i. uninfected animals (Naïve; open diamonds) served as negative controls. Numbers of analyzed animals are given in parentheses. Means (black bars) and levels of significance (*P*-values) determined by the Mann-Whitney-U test are indicated. Data shown were pooled from three independent experiments.

We further assessed the immunopathological responses of mice upon infection with the *C. jejuni* knockout mutant NCTC11168::*cj0268c*. Irrespective of the strain, gnotobiotic mice displayed comparable histopathological changes in hematoxylin and eosin (H&E) stained colonic paraffin sections at day 6 p.i. (not shown). Given that apoptosis is a commonly used diagnostic marker in the histopathological evaluation and grading of intestinal disease [21] and a key feature of *C. jejuni* induced ulcerative enterocolitis in gnotobiotic IL-10^−/−^ mice [18], we quantitatively assessed caspase-3^+^ cells within the colonic mucosa following infection with the respective *C. jejuni* strains. Six days upon peroral challenge, mice infected with the mutant strain NCTC11168::*cj0268c* displayed significantly less distinct colonic epithelial apoptosis when compared to wildtype and complemented controls as indicated by approximately 35% lower caspase-3^+^ positive cell numbers in the colonic mucosa of the former (p<0.05; **Fig. 5A**).

Given that recruitment of pro-inflammatory immune cell populations to sites of inflammation is a hallmark of human campylobacteriosis [21], we next quantitatively assessed the influx of innate and adaptive immune as well as effector cell populations into the large intestine by applying *in situ* immunohistochemical staining of colonic paraffin sections. Following *C. jejuni* infection, a marked influx of CD3^+^ cells (i.e. T lymphocytes) into the colonic mucosa and lamina propria could be detected until day 6 p.i. (**Fig. 5B**). This increase, however, was significantly less pronounced in mice infected with the knockout mutant NCTC11168::*cj0268c* as compared to parental strain NCTC11168 and complemented strain NCTC11168::*cj0268c*-comp-*cj0268c* infected control animals (p<0.01 and p<0.05, respectively; **Fig. 5B**). Irrespective of the *C. jejuni* strain, infected mice displayed comparable increases of Foxp3^+^ regulatory T cells, B220^+^ B lymphocytes, MPO7^+^ neutrophils, and F4/80^+^ macrophages and monocytes in the colonic mucosa at day 6 p.i. as compared to naïve animals (p<0.0005–0.0001; **Fig. 5C–F**).

**Figure 6:**
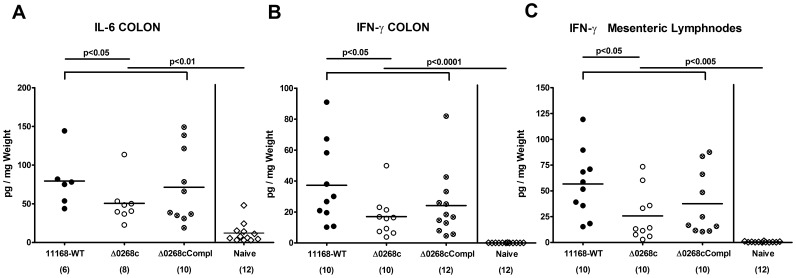
Impact of the *cj0268c* gene on intestinal pro-inflammatory cytokine responses following infection of gnotobiotic IL-10^−/−^ mice. Gnotobiotic IL-10^−/−^ mice were generated by antibiotic gut decontamination and perorally infected with *C. jejuni* NCTC11168 (11168-WT, closed circles), mutant strain NCTC11168::*cj0268c* (Δ0268c, open circles), or the complemented strain NCTC11168::*cj0268c*-comp-*cj0268c* (Δ0268cCompl, crossed circles) as described (see methods). Colonic (A) IL-6 and (B) IFN-γ levels as well as IFN-γ secretion in (C) mesenteric lymphnodes (MLNs) were determined in culture supernatants of *ex vivo* biopsies taken from the respective organs at day 6 p.i. Uninfected animals (naïve; open diamonds) served as negative controls. Numbers of analyzed animals are given in parentheses. Means (black bars) and levels of significance (*P*-values) determined by the Mann-Whitney-U test are indicated. Data shown were pooled from three independent experiments.

We next determined intestinal pro-inflammatory cytokine expression levels upon *C. jejuni* infection. Lower colonic apoptotic cell and T lymphocyte counts were accompanied by lower IL-6 and IFN-γ protein concentrations in *ex vivo* colonic biopsies obtained from gnotobiotic IL-10^−/−^ mice six days following infection with the knockout mutant NCTC11168::*cj0268c* as compared to the parental strain NCTC11168 (**Fig. 6A, B**). The impact of *cj0268c* in mediating *C. jejuni* induced immunopathology was further underlined by lower IFN-γ levels in *ex vivo* biopsies of draining mesenteric lymphnodes in mutant strain NCTC11168::*cj0268c* as compared to parental strain NCTC11168 infected mice (p<0.05; **Fig. 6C**). Whereas intestinal pro-inflammatory cytokine levels in complemented and wildtype strain infected mice did not differ, a trend towards higher intestinal IL-6 and IFN-γ concentrations six days following complemented as compared to knock-out mutant strain infection could be observed. Given high standard deviations in the respective groups, however, the differences did not reach statistical significance (**Fig. 6**).

Taken together, *cj0268c* gene deficiency does not alter *C. jejuni* NCTC11168 colonization capacities *in vivo*. In addition, the Cj0268*c* protein is involved in mediating *C. jejuni* induced acute enteritis as indicated by i.) less shrinkage of the small as well as large intestines, ii.) less abundance of colonic epithelial apoptotic cells, iii.) less distinct T lymphocyte infiltrations in the colonic mucosa and iv.) less pro-inflammatory cytokine secretion at intestinal tissue sites including mesenteric lymphnodes of gnotobiotic IL-10^−/−^ mice infected with the knockout mutant strain NCTC11168::*cj0268c* when compared to control animals.

## Discussion

We have recently shown that the *C. jejuni* protein Cj0268c is an important prerequisite for pathogen adhesion and invasion of host cells *in vitro* [17]. In the present study we investigated the impact of *cj0268c* in *C. jejuni* induced immunopathology *in vivo*. To prevent conventionally colonized IL-10^−/−^ mice from spontaneous chronic colitis due to antigenic stimuli derived from the intestinal microbiota, mice were subjected to at least 3 months broad-spectrum antibiotic treatment starting immediately after weaning [18]. Upon peroral *C. jejuni* infection gnotobiotic IL-10^−/−^ mice develop non-selflimiting ulcerative enterocolitis within one week p.i. mimicking severe campylobacteriosis in immuno-compromized patients [18]. Here, kinetic analyses revealed that until day 6 following peroral infection, mice harboured high intestinal loads of the knockout mutant strain NCTC11168::*cj0268c*, which were comparable to those detected in mice upon infection with the parental strain NCTC11168 or the complemented version NCTC11168::*cj0268c*-comp-*cj0268c*. Hence, knockout of the *cj0268c* gene did neither compromise infection capacities *in vitro* [17] nor *in vivo*. Of note, genetic complementation clearly demonstrates that the generated NCTC11168::*cj0268c* knock-out mutant strain is not polar. Remarkably, mice infected with the mutant strain NCTC11168::*cj0268c* displayed significantly less severe immunopathology in the intestinal tract as compared to mice infected with the parental strain NCTC11168 or the complemented *C. jejuni* strain NCTC11168::*cj0268c*-comp-*cj0268c* as indicated by a plethora of results. First, knockout mutant strain NCTC11168::*cj0268c* infected gnotobiotic IL-10^−/−^ mice displayed less shrinkage of the small as well as large intestines which is a rather rough, but reliable indicator for less pronounced intestinal pathology [20], [22], [23], [24]. Second, this was further supported by less abundance of caspase-3^+^ cells in the colonic mucosa given that apoptosis is a commonly used diagnostic marker in the histopathological evaluation and grading of intestinal disease [21] and a key feature of *C. jejuni* induced ulcerative enterocolitis in gnotobiotic IL-10^−/−^ mice [18]. Third, T lymphocytes well known to play a pivotal role in induction and perpetuation of *C. jejuni* induced immunopathology in mice [18], [21], [25], [26], [27] were infiltrating the intestinal mucosa and lamina propria of gnotobiotic IL-10^−/−^ mice following infection with knockout mutant NCTC11168::*cj0268c* to a far lesser extent as compared to the applied control strains. Fourth, virtually no translocation of viable *C. jejuni* from the intestinal tract to MLNs was observed upon infection with mutant strain NCTC11168::*cj0268c*. Fifth, expression of pro-inflammatory cytokines such as IL-6 and IFN-γ was more than 50% lower in *ex vivo* biopsies derived from colon and MLNs upon infection with the mutant versus the parental strain. In our previous work where we independently studied the *C. jejuni*-induced immunopathological sequelae in two other murine *C. jejuni* infection models we could unequivocally demonstrate that severity of campylobacteriosis was paralleled by up-regulated expression levels of IFN-γ and IL-6 in both, the colon and MLNs [18], [21], [25], further supporting significance of the results presented here. It is tempting to speculate that the decreased intestinal IFN-γ and IL-6 levels following mutant as compared to parental strain infection might be indicative for shifted intestinal T cell populations in the absence of *cj0268c* which needs to further unraveled.

Irrespective of the *C. jejuni* strain, however, infected gnotobiotic IL-10^−/−^ mice developed comparable clinical symptoms of enteritis over time in the presented study, which was contrasting the less pronounced immunopathological outcome in the intestinal tract. Despite the observation of comparable clinical symptoms upon infection with the knockout mutant strain NCTC11168::*cj0268c*, one needs to take into account that the clinical picture of a disease is rather the sum of different effects resulting from several levels of immunopathological mechanisms. Furthermore, the *cj0268c* gene is by far not the only factor involved in adhesion and invasion and subsequent induction of immunopathology [28]. Nevertheless, severity of *C. jejuni* induced enteritis can vary considerably between infected human individuals and range from very mild, sublatent and self-limiting complaints to severe symptoms such as abdominal cramps, fever, and bloody diarrhea depending on the dysbalance between the immune status of the host and the respective pathogenicity factors of the pathogen expressed in parallel [29].

One needs to take further into account, that *C. jejuni* infection in the *in vivo* infection model applied here results in a devastating outcome, namely non-selflimiting acute ulcerative enterocolitis leading to death within 10 days [18]. Hence, if any beneficial effect is observed in such a hyper-acute model system, the biological relevance gets more plausible. Furthermore, our *in vitro* results revealed that adhesive properties of the mutant strain were not 100%, but reached rather 60% [17]. Moreover, we have recently shown in different murine infection models that Toll-like-receptor (TLR)-4 dependent signalling of *C. jejuni* lipooligosaccharide is a key factor in *C. jejuni* induced immunopathology as indicated by ameliorated clinical and intestinal immunopathology in *C. jejuni* infected gnotobiotic TLR-4 deficient as well as IL-10 deficient mice lacking TLR-4 [18], [21], [30].

Taken together, our previous and actual results have shown that *cj0268c* is involved in *C. jejuni* adhesion and invasion of vertebrate cells subsequently inducing significant immunopathology in the host with varying clinical features. Due to the lack of appropriate animal models in the past, the impact of most of the so far identified pathogenicity factors of *C. jejuni* involved in pathogen-host-interaction and thus their biological relevance in inducing campylobacteriosis have not been investigated *in vivo* yet.

In conclusion, future *in vivo* studies should further unravel the distinct molecular mechanisms and orchestration of different pathogenicity factors contributing to *C. jejuni* induced disease.

## Materials and Methods

### Ethics Statement

All animal experiments were conducted according to the European Guidelines for animal welfare (2010/63/EU) with approval of the commission for animal experiments headed by the “Landesamt für Gesundheit und Soziales” (LaGeSo, Berlin, Germany; registration numbers G0123/12). Animal welfare was monitored twice daily by assessment of clinical conditions.

### Mice

IL-10^−/−^ mice (in C57BL/10 background, B10) were bred and maintained in the facilities of the “Forschungsinstitut für Experimentelle Medizin” (FEM, Charité - Universitätsmedizin, Berlin, Germany), under specific pathogen-free (SPF) conditions.

To eradicate the commensal gut flora, mice were transferred to sterile cages and treated by adding ampicillin (1 g/L; Ratiopharm), vancomycin (500 mg/L; Cell Pharm), ciprofloxacin (200 mg/L; Bayer Vital), imipenem (250 mg/L; MSD), and metronidazole (1 g/L; Fresenius) to the drinking water *ad libitum* as described earlier [20] starting at 3 weeks of age right after weaning. Age matched female mice were subjected to the quintuple antibiotic treatment for 3–4 months before the infection experiment.

### *C. jejuni* Infection of Mice

Mice were infected with approximately 10^9^ viable CFU of *C. jejuni* strains NCTC11168 (parental strain), the *C. jejuni* mutant strain NCTC11168::*cj0268c* (lacking the *cj0268c* gene [17]), or its complemented version NCTC11168::*cj0268c*-comp-*cj0268c* [17], respectively, by gavage in a total volume of 0.3 mL PBS on two consecutive days (day 0 and day 1).

### Clinical Score

To assess clinical signs of *C. jejuni* induced infection on a daily basis, a standardized cumulative clinical score (maximum 12 points, addressing the occurrence of blood in feces (0 points: no blood; 2 points: microscopic detection of blood by the Guajac method using Haemoccult, Beckman Coulter/PCD, Krefeld, Germany; 4 points: overt blood visible), diarrhea (0: formed feces; 2: pasty feces; 4: liquid feces), and the clinical aspect (0: normal; 2: ruffled fur, less locomotion; 4: isolation, severely compromized locomotion, pre-final aspect) was used [18].

### Sampling Procedures

Mice were sacrificed by isofluran treatment (Abbott, Germany). Cardiac blood and tissue samples from mesenteric lymphnodes, spleen, liver, kidney and GI tract (stomach, duodenum, ileum, colon) were removed under sterile conditions. Absolute small and large intestinal lengths were determined by measuring the distances from the transition of the stomach to the duodenum to the very distal terminal ileum and from the ascending colon leaving the caecum to the rectum, respectively, by a ruler and expressed in cm. GI samples from each mouse were collected in parallel for immunohistochemical, microbiological, and immunological analyses. Immunohistopathological changes were determined in colonic samples immediately fixed in 5% formalin and embedded in paraffin. Sections (5 µm) were stained with H&E or respective antibodies for *in situ* immunohistochemistry.

### Immunohistochemistry

*In situ* immunohistochemical analysis of colonic paraffine sections was performed as described previously [18], [21], [25], [31]. Primary antibodies against cleaved caspase-3 (Asp175, Cell Signaling, USA, 1∶200), CD3 (#N1580, Dako, Denmark, dilution 1∶10), myeloperoxidase-7 (MPO-7, # A0398, Dako, 1∶10000), F4/80 (# 14-4801, clone BM8, eBioscience, 1∶50), Foxp3 (FJK-16s, eBioscience, 1∶100), and B220 (eBioscience, San Diego, CA, USA, 1∶200) were used. For each animal, the average number of positively stained cells within at least six high power fields (HPF, 0.287 mm^2^; 400 × magnification) were determined microscopically by three independent investigators.

### Quantitative Analysis of *C. jejuni* (Translocation)

Live *C. jejuni* were detected in feces or at time of necropsy (day 6 p.i.) in luminal samples taken from the stomach, duodenum, ileum, or colon dissolved in sterile PBS by culture as described earlier [18], [21]. To quantify bacterial translocation, MLNs, spleen, liver (≈1 cm^2^) and kidney were homogenized in sterile PBS and analyzed by cultivating on karmali agar (Oxoid, Wesel, Germany) in a microaerophilic atmosphere at 37°C for at least 48 hours. Cardiac blood (≈200 µL) was directly streaked onto karmali agar and cultivated accordingly. The respective weights of fecal or tissue samples were determined by the difference of the sample weights before and after asservation. The detection limit of viable pathogens was ≈100 CFU per g.

### Cytokine Detection in Culture Supernatants of *ex vivo* Biopsies taken from Colon and Mesenteric Lymphnodes

Colon biopsies were cut longitudinally, and washed in PBS. Mesenteric lymphnodes or strips of approximately 1 cm^2^ colon tissue were placed in 24-flat-bottom well culture plates (Nunc, Wiesbaden, Germany) containing 500 µL serum-free RPMI 1640 medium supplemented with penicillin (100 U/mL) and streptomycin (100 µg/mL; PAA Laboratories). After 18 h at 37°C, culture supernatants were tested for IL-6 and IFN-γ by the Mouse Inflammation Cytometric Bead Assay (CBA; BD Biosciences) on a BD FACSCanto II flow cytometer (BD Biosciences).

### Statistical Analysis

Mean values, medians, and levels of significance were determined using Mann-Whitney-U test. Two-sided probability (*P*) values ≤0.05 were considered significant. All experiments were repeated at least twice.
